# LED lighting and exogenous cytokinin enhance budbreak and winter growth of ‘Washington’ navel orange in the nursery

**DOI:** 10.3389/fpls.2025.1735154

**Published:** 2025-12-16

**Authors:** Deived Uilian de Carvalho, Rayane Barcelos Bisi, Kim D. Bowman, Ute Albrecht

**Affiliations:** 1 Southwest Florida Research and Education Center, Horticultural Sciences Department, Institute of Food and Agricultural Sciences, University of Florida, Immokalee, FL, United States; 2 U.S. Horticultural Research Laboratory, Agricultural Research Service, United States Department of Agriculture, Ft. Pierce, FL, United States

**Keywords:** citrus propagation, photoperiod control, light spectra, 6-benzylaminopurine (BA), rootstock cultivars, bud growth and development, nursery productivity

## Abstract

Citrus nurseries typically reduce budding activity during winter due to weak growth from short photoperiods and low temperatures. Modern light-emitting diodes (LEDs) and exogenous application of plant growth regulators (PGRs), such as the synthetic cytokinin 6-benzylaminopurine (BA), may mitigate these winter challenges by extending daylength and stimulating budbreak. We report in this paper the effects of different LED spectra and BA on budbreak and growth of ‘Washington’ navel orange (*Citrus* × *sinensis*) grafted on two rootstocks during winter. A 4 × 2 × 2 factorial design was used, including four light levels (NoSL, no supplemental light; FSL, full supplemental light from budding to 12 weeks after budding (wab); BWSL, blue and white supplemental light from budding to 12 wab; and BWSL-FSL, BWSL from budding to 6 wab changing to FSL from 6 to 12 wab), two rootstocks (Carrizo citrange, *C.* × *sinensis* × *Poncirus trifoliata*; and Rubidoux trifoliate orange, *P. trifoliata*), and two PGR levels (NoBA and BA). Each treatment was replicated six times with 24 plants per replicate, totaling 2304 experimental plants. Different horticultural responses were assessed over time. Both FSL and BWSL-FSL significantly improved budbreak (79% and 77%, respectively) and survival (80% and 77%, respectively) compared to NoSL (62% and 64%, respectively). Budbreak was 1.3-fold higher on Carrizo than on Rubidoux. When supplemented with FSL, Carrizo produced the largest rootstock diameter (7.80 mm), scion diameter (4.37 mm), total leaf area (678 cm²), and scion dry biomass (7.87 g), representing a 5.7-fold increase compared to Rubidoux without supplemental light (1.38 g). Carrizo plants supplemented with FSL had more nodes (16) and longer internodes (34.5 mm) compared to NoSL (10 nodes and 17.5 mm, respectively) and BWSL (13 nodes and 25.4 mm, respectively). BA application improved horticultural responses regardless of rootstock, and the combination of BWSL and BA enhanced chlorophyll content compared to most of the other treatment combinations. These findings confirm the effectiveness of tailored LED light, suitable rootstock selection, and PGR strategies in optimizing citrus nursery production and tree quality during the winter months when natural light is limited.

## 1 Introduction

Citrus nurseries normally plan for a significant reduction in budding activities during the winter months because of greatly reduced tree growth. This seasonal decline in growth is primarily attributed to short photoperiods, limited light intensities, and low temperatures (Borchert et al., 2015; Davis and Burns, 2016). This constrains the development of both citrus rootstock liners and buddings (Brar and Spann, 2014; Bowman and Albrecht, 2021), leading to decreased nursery productivity and profitability. For instance, even slight changes in photoperiod can lead to substantial advances or delays in physiological processes related to bud development (Mawphlang and Kharshiing, 2017). Without supplemental lighting and/or horticultural interventions such as plant growth regulators (PGRs), nurseries face considerable challenges in maintaining plant growth during this period.

Light-emitting diodes (LEDs) have become a highly efficient and versatile technology for plant cultivation, particularly under low-light or winter conditions (Rehman et al., 2017; Paradiso and Proietti, 2022). Their spectral flexibility allows precise control of light quality and intensity, influencing plant photosynthesis, morphology, and metabolism (Christie et al., 2015; Bantis et al., 2018). In citrus nurseries, recent studies have demonstrated that extending daylength with LED lighting can enhance growth of young trees during the winter months (Bowman and Albrecht, 2021). Among light spectra, blue wavelengths contribute significantly to vegetative growth by promoting leaf expansion, shoot elongation, and chlorophyll synthesis (Li et al., 2017; Naznin et al., 2019). Full- or broad-spectrum LEDs, which better approximate natural sunlight, can further improve photosynthetic performance and overall plant vigor, though their effects vary depending on species and growth conditions (Mitchell et al., 2015; Ma et al., 2021).

Another strategy to enhance bud development is the application of PGRs. Cytokinin, the primary hormone regulating bud outgrowth, promotes budbreak, particularly in environments with limiting growth conditions (Ni et al., 2017). This hormone is particularly important in citrus nursery plants during winter, when cold temperatures reduce growth. Synthetic cytokinin, such as 6-benzylaminopurine (BA), has been shown to overcome these winter limitations by stimulating cell division and enhancing bud initiation. Previous studies have demonstrated the effectiveness of BA in promoting budbreak in satsuma mandarin (*Citrus unshiu* Marc.; Zhu et al., 1989) and apple (*Malus × domestica* Borkh.) trees (Wertheim and Estabrooks, 1994; McArtney and Obermiller, 2015). Its application during winter supports the timely emergence of buds and may also help stimulate plant vigor and productivity, as observed in a recent citrus study (Niedz and Bowman, 2023). By leveraging BA, citrus nurseries can better regulate growth cycles, ensuring more consistent and robust budbreak even under adverse winter conditions.

The winter dormancy induced by trifoliate orange (*Poncirus trifoliata* (L.) Raf.) type rootstocks is another important factor that limits tree growth in citrus nurseries (Yelenosky, 1985; Primo-Millo and Agustí, 2020). This dormancy results from their deciduous nature and sensitivity to the photoperiod (Brar and Spann, 2014). Trifoliate orange and different hybrids of trifoliate are widely used in Florida, accounting for approximately 80% of the commercial rootstocks used in the state (Castle et al., 2020), as well as in California and other citrus-growing regions. This dormancy trait may influence how different trifoliate-type rootstocks respond to supplemental light and other growth-enhancing practices.

Research is needed to optimize light strategies for citrus nursery production, particularly to identify the most effective and economical combinations of light spectra that promote optimal budbreak, plant growth, and health during winter. To address these challenges, supplemental light, applied at different spectra and combined with BA, may be employed in citrus nurseries to extend daylength, promote budbreak and growth, and mitigate the effects of shorter winter days. Building on our previous work, the present study aimed to investigate the effects of these factors on enhancing budbreak and shoot flush growth in ‘Washington’ navel orange budded on Carrizo citrange (*C.* × *sinensis* (L.) Osbeck × *P. trifoliata*) and Rubidoux trifoliate orange (*P. trifoliata*) during winter. The results from this study offer valuable insights for improving citrus growth performance and profitability during the winter months, ultimately advancing nursery practices and contributing to the sustainability of the citrus industry.

## 2 Materials and methods

### 2.1 Plant materials

‘Washington’ navel orange (*Citrus* × *sinensis* (L.) Osbeck) plants budded on Carrizo citrange and Rubidoux trifoliate orange were used in this study. All rootstock seedlings were grown from seeds in sterilized soilless potting medium composed of 75-85% peat moss and 10-20% perlite (Pro-Mix BX, Premier Horticulture, Inc., Quakertown, PA, United States). Rootstock seeds were sown into 3.8 cm × 21.0 cm cone-tainers (Stuewe and Sons, Inc., Tangent, OR, USA) containing the potting medium (one seed per container). Any off-type seedlings were identified by morphological characteristics and discarded (Bisi et al., 2020). After 3–4 months of growth, true-to-type seedlings were transplanted into 2.54-L pots (Treepots; Stuewe and Sons, Inc., Tangent, OR, USA) filled with the same soilless potting medium. Two weeks before budding, the stem diameters of the rootstock seedlings were measured 15 cm above the potting medium to ensure that the seedlings had an appropriate diameter for grafting. Seedlings with smaller stem diameters and/or any other growth abnormality were excluded from the experiment. Rootstock seedlings were sorted by diameter size and then randomly assigned to each treatment group. This procedure ensured a uniform distribution of rootstock liner sizes across all treatment groups.

On November 14, 2023, the six-month-old rootstock liners were budded with the sweet orange clone ‘Washington’ navel FDACS DPI-05-28–51 using the inverted T method. After budding, all grafted buds were wrapped with budding tape for three weeks. Budded plants were arranged on the greenhouse benches so that the grafted buds faced southward to promote vegetative budbreak and scion shoot growth (Niedz and Bowman, 2023). Four weeks after budding (wab), the rootstock was trimmed at 65 cm height and looped to force bud growth, as previously described by Bowman (1999).

### 2.2 Supplemental light and 6-benzylaminopurine treatments

**Figure 1:**
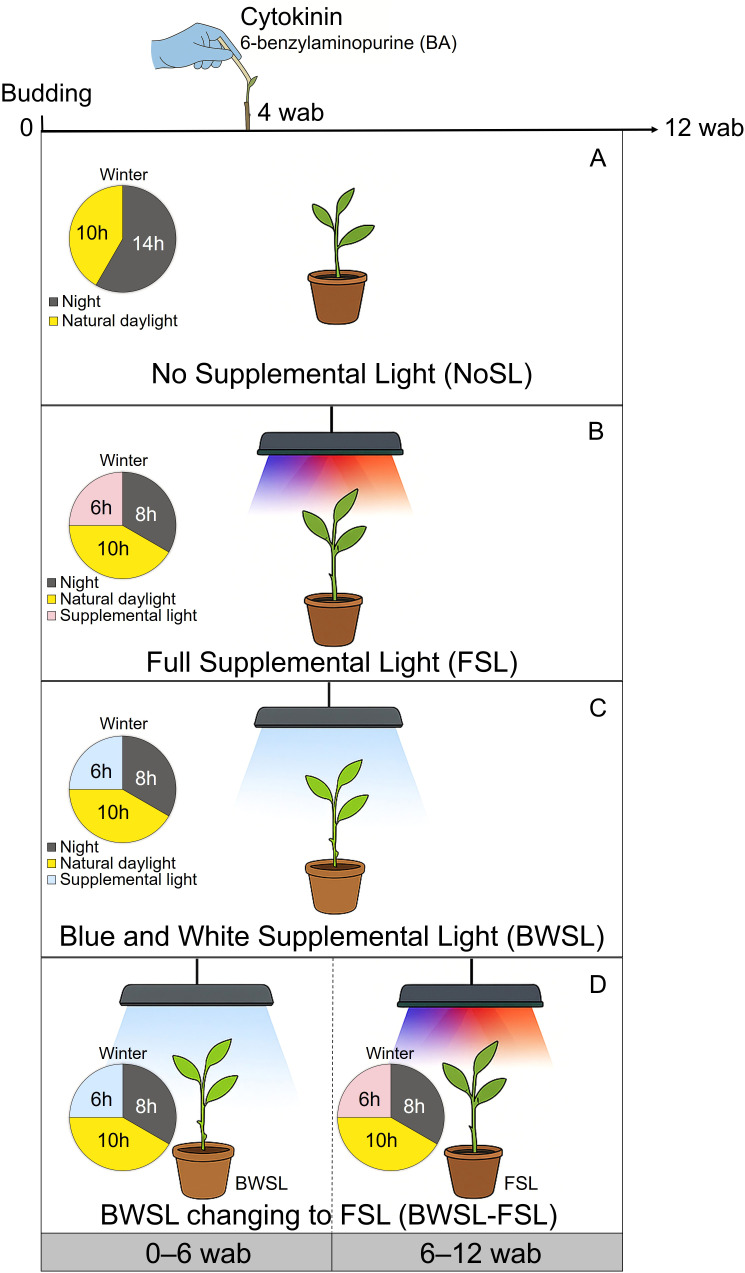
Schematic representation of supplemental light treatments used to extend the photoperiod to 16 h during winter in a temperature-controlled greenhouse for ‘Washington’ navel orange plants grafted onto Carrizo citrange and Rubidoux trifoliate orange rootstocks. Control plants received only natural daylight (NoSL, no supplemental light) **(A)**. Full-spectrum supplemental light (FSL) used from budding (0) to 12 weeks after budding (wab) **(B)**. Blue and white supplemental light (BWSL) used from 0 to 12 wab **(C)**. Combined treatment (BWSL-FSL): BWSL from 0 to 6 wab changing to FSL from 6 to 12 wab **(D)**.

All plants received natural light during the day, and some received supplemental LED light at the end of the night hours to extend daylength to 16 hours (Bisi et al., 2024). The light sources used in this study were Elixia 600W LEDs (Heliospectra, Göteborg, Sweden). The fixture used had LED elements used in the following two combinations: 1) full supplemental light – FSL (blue, peak at 450 nm; red, peak at 660 nm; far-red, peak at 735 nm; and white 5700K, peaks at 446 nm, 534 nm, and 625 nm); and 2) blue (peak at 450 nm) combined with white (5700K, peaks at 446 nm, 534 nm, and 625 nm) supplemental light – BWSL. Lights were supplied from budding to 12 wab. A third light treatment using BWSL from budding to 6 wab and changing to FSL from 6–12 wab was also tested in this study (BWSL-FSL). All light elements were set to full intensity when they were active. Control plants were not supplemented with LED light, relying on natural sunlight only (NoSL, no supplemental light). A summary of the supplemental light treatments is shown in **Figure 1**.

The light fixtures were positioned at a height of 110 cm above the bud insertion to ensure consistent distribution of photosynthetic photon flux density (PPFD) across all plants subjected to different lights. To accommodate natural fluctuations in daylength throughout the 12-week study period, the timing of supplemental light was modified; whenever there was a 15-minute shift in natural daylight duration, the timing of the LED was adjusted to maintain a consistent 16-hour daylength. Light energy was measured in each treatment using a spectrometer (PG100N, United Power Research Technology Corp., Zhunan Township, Taiwan) positioned at 110 cm below the light fixture, at the level of bud insertion. Measurements were taken at night under moonless conditions to accurately determine the average PPFD provided by each LED fixture.

**Figure 2:**
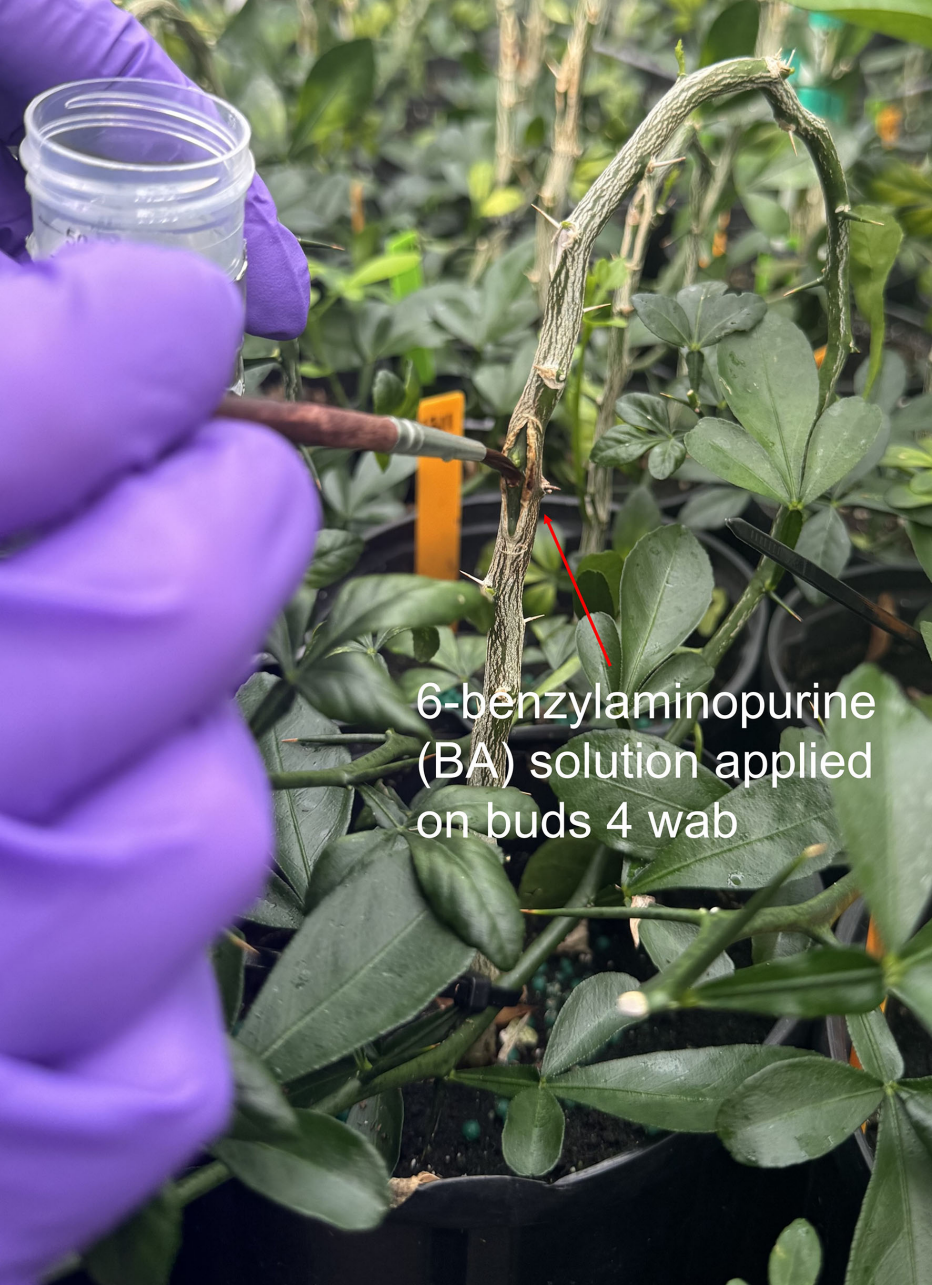
At four weeks after budding (wab), rootstock liners were looped to promote bud growth, and buds received 6-benzylaminopurine (BA) application with a micro brush.

Four weeks after budding (wab; **Figure 1**), half of the budded plants (*n* = 576 plants per rootstock) were treated with a 5.0 mM 6-benzylaminopurine (BA, PhytoTech Labs, Inc., Lenexa, KS, USA) solution (Niedz and Bowman, 2023). The BA solution was applied with a micro brush (Testor Corp., Rockford, IL, USA) directly over each bud (**Figure 2**). The other half of the budded plants were not treated with BA (*n* = 576 plants per rootstock). The experimental design was completely randomized in a 4 × 2 × 2 factorial arrangement with four levels of supplemental light (NoSL, FSL, BWSL, and BWSL-FSL), two rootstocks (Carrizo and Rubidoux), and two levels of PGRs (NoBA and BA). Each combination (*n* = 16) was replicated six times using 24 plants as experimental units. A total of 2304 plants were assessed in this study.

### 2.3 Plant care and maintenance

Plants were grown in a temperature-controlled greenhouse, where the ambient temperature was maintained between 21 and 27 °C. This temperature range was controlled with forced-air heaters, water-cooled pads, ventilation fans, and circulating fans. The greenhouse was covered with an 8.0-mm twin-wall polycarbonate panel (Lexan™ Thermoclear 15, General Electric Co., Norcross, GA, USA).

Plants were irrigated and fertilized weekly until runoff using water-soluble fertilizer, a solution of Peters Professional 20N-10P-20K formulation (The Scotts Company, Marysville, OH, USA) at a concentration of 400 mg N/L. Additional water between fertilization was applied to the experimental plants as required. Insecticides and miticides were sprayed as needed to ensure plant health. The air temperature was monitored every 10 minutes in each treatment combination from budding to 12 wab, using a HOBO data logger (MX2203, Onset Computer Corp., Bourne, MA, USA).

Photosynthetic photon flux density (PPFD), which quantifies the instantaneous flux of photons within the photosynthetically active radiation (PAR) waveband (400–700 nm), was measured for each lighting treatment using analog full-spectrum quantum sensors (SQ-500, Apogee Instruments, Logan, UT, USA). Each sensor was connected to a data logger, and PPFD values were recorded every 150 s and averaged every 900 s throughout the experiment. These measurements were then used to calculate the daily light integral (DLI), representing the total number of photosynthetically active photons received per unit area per day, using the following formula:


$$\text{DLI}=\frac{\sum \text{PPFD}\times 900}{1,000,000},$$

where DLI is expressed in mol m^-2^ day^-1^ and PPFD represents the photosynthetically active radiation measured in µmol m^-2^ s^-1^.

### 2.4 Vegetative growth assessments

Plants were assessed for budbreak (bud growth initiation: bud shoot tip at least 2.0 mm in length) and scion shoot growth (shoot growth length) at 6, 9, and 12 wab. At 12 wab, scion stem diameters were measured 5.0 cm above the graft union, and rootstock stem diameters were measured 15 cm above the potting medium for each budded plant. The number of nodes and the internode length were assessed up to the last fully expanded leaf, and the total leaf area was determined. Leaf area was measured using a Li-Cor Li-3100C leaf area meter (Li-COR Biosciences, Lincoln, NE, USA). Chlorophyll content was determined at 12 wab by measuring the first fully expanded leaf of each budded plant using a portable chlorophyll meter (SPAD-502, Minolta, Spectrum Technologies, Inc., Plainfield, IL, USA). Leaf temperatures were recorded at night, at 12 wab, with an infrared radiometer (MI-210, Apogee Instruments, Logan, UT, USA) on the first fully expanded leaf from the scion shoots of nine plants per combination. At 12 wab, the scion shoot was excised and dried at 70 °C in an oven chamber until constant weight to determine the total biomass dry weight (g).

### 2.5 Statistical analysis

The experiment was conducted as a 4 × 2 × 2 factorial, with four light levels (NoSL, FSL, BWSL, and BWSL-FSL), two rootstocks (Carrizo and Rubidoux), and two PGR levels (NoBA and BA). Data were tested for normality using the Shapiro-Wilk test and for homogeneity of variances using Bartlett’s test at p ≤ 0.05. Vegetative measurements were analyzed using a three-way analysis of variance (ANOVA). Average temperatures (maximum, minimum, and mean) and daily light integrals (DLIs) were analyzed using a two-way ANOVA, considering light and time after budding as factors: four light levels and three levels of time after budding (0–6, 6–9, and 9–12 wab). Mean differences were evaluated with Tukey’s Honestly Significant Difference (HSD) test at α = 0.05.

For hierarchical clustering and heatmap analyses, all variables were averaged at 12 wab for each treatment. Values were standardized, and clustering was performed using Euclidean distances with complete linkage (Ward’s method). Cluster robustness was evaluated via 10,000 bootstrap resamplings, and Approximately Unbiased (AU) p-values were calculated; clusters with AU-values closer to 100% are considered more stable. All analyses were performed in R v. 4.4.0 (The R Foundation for Statistical Computing, Vienna, Austria) using the RStudio interface and the agricolae, pvclust, and pheatmap packages.

## 3 Results

### 3.1 Budbreak and survival

The budbreak percentages of ‘Washington’ navel buds were significantly influenced by light (p ≤ 0.01), rootstock (p ≤ 0.001), and PGR (p ≤ 0.01) over the evaluated period from 6 to 12 wab (**Table 1**). However, there was no significant interaction among these factors; therefore, only the main effects are presented in **Table 1**. Among the light levels, FSL and BWSL-FSL induced the highest percentages at all times. By 12 wab, these treatments achieved 78.5% and 76.9% bud outgrowth, respectively, while the NoSL control resulted in only 62.1% budbreak (p ≤ 0.01). Carrizo induced a higher budbreak than Rubidoux, with a 30% increase compared to Rubidoux by the end of the experiment (p ≤ 0.001). A similar trend was observed in response to PGR use, where BA increased budbreak by an average of 11% at 12 wab (p ≤ 0.01) compared to NoBA.

**Table 1:** Percentage of vegetative budbreak (%) at 6, 9, and 12 weeks after budding (wab) and bud survival at 12 wab of ‘Washington’ navel orange nursery plants grafted on Carrizo citrange and Rubidoux trifoliate orange rootstocks grown under supplemental LED lights and treated with 6-benzylaminopurine (BA) during the winter months.

Factor	Budbreak (%)			Bud survival (%)
	6 wab	9 wab	12 wab	12 wab
Light				
NoSL	54.5 b	58.3 b	62.1 b	64.1 b
FSL	70.5 a	75.9 a	78.5 a	80.2 a
BWSL	62.8 ab	69.1 ab	72.7 ab	73.4 ab
BWSL-FSL	69.1 a	74.5 a	76.7 a	77.4 a
Rootstock				
Carrizo	75.9 a	78.9 a	82.1 a	83.9 a
Rubidoux	52.6 b	59.9 b	62.9 b	63.7 b
PGR				
NoBA	53.1 b	63.4 b	68.8 b	70.7 b
BA	75.3 a	75.5 a	76.2 a	76.9 a
p-value				
Light	**	**	**	**
Rootstock	***	***	***	***
PGR	***	***	**	*
Light × Rootstock	0.201	0.309	0.424	0.503
Light × PGR	0.917	0.853	0.659	0.395
Rootstock × PGR	0.055	0.945	0.917	0.773
Light × Rootstock × PGR	0.964	0.799	0.827	0.762

NoSL, no supplemental light from 0 to 12 wab; FSL, full supplemental light from 0 to 12 wab; BWSL, blue and white supplemental light from 0 to 12 wab; BWSL-FSL, blue and white supplemental light from 0 to 6 wab changing to full supplemental light from 6 to 12 wab; NoBA, no 6-benzylaminopurine; 6-BA, 6-benzylaminopurine. Different letters within columns indicate significant differences between light treatments, rootstocks, and plant growth regulators at each assessment according to Tukey’s honestly significant difference (HSD) test (α = 0.05). Level of significance: *, p ≤ 0.05; **, p ≤ 0.01; ***, p ≤ 0.001.

Differences in bud survival at 12 wab were also evident among the main effects, although no interactions were detected between the factors (**Table 1**). Plants grown under supplemental light had a higher bud survival compared to the NoSL control, particularly with FSL and BWSL-FSL, which induced a 23% increase over NoSL (p ≤ 0.01). The choice of rootstock and PGR application also proved to be effective in enhancing bud survival, with Carrizo (p ≤ 0.001) and BA (p ≤ 0.05) inducing improvements of 32% and 9%, respectively.

### 3.2 Scion shoot growth

**Figure 3:**
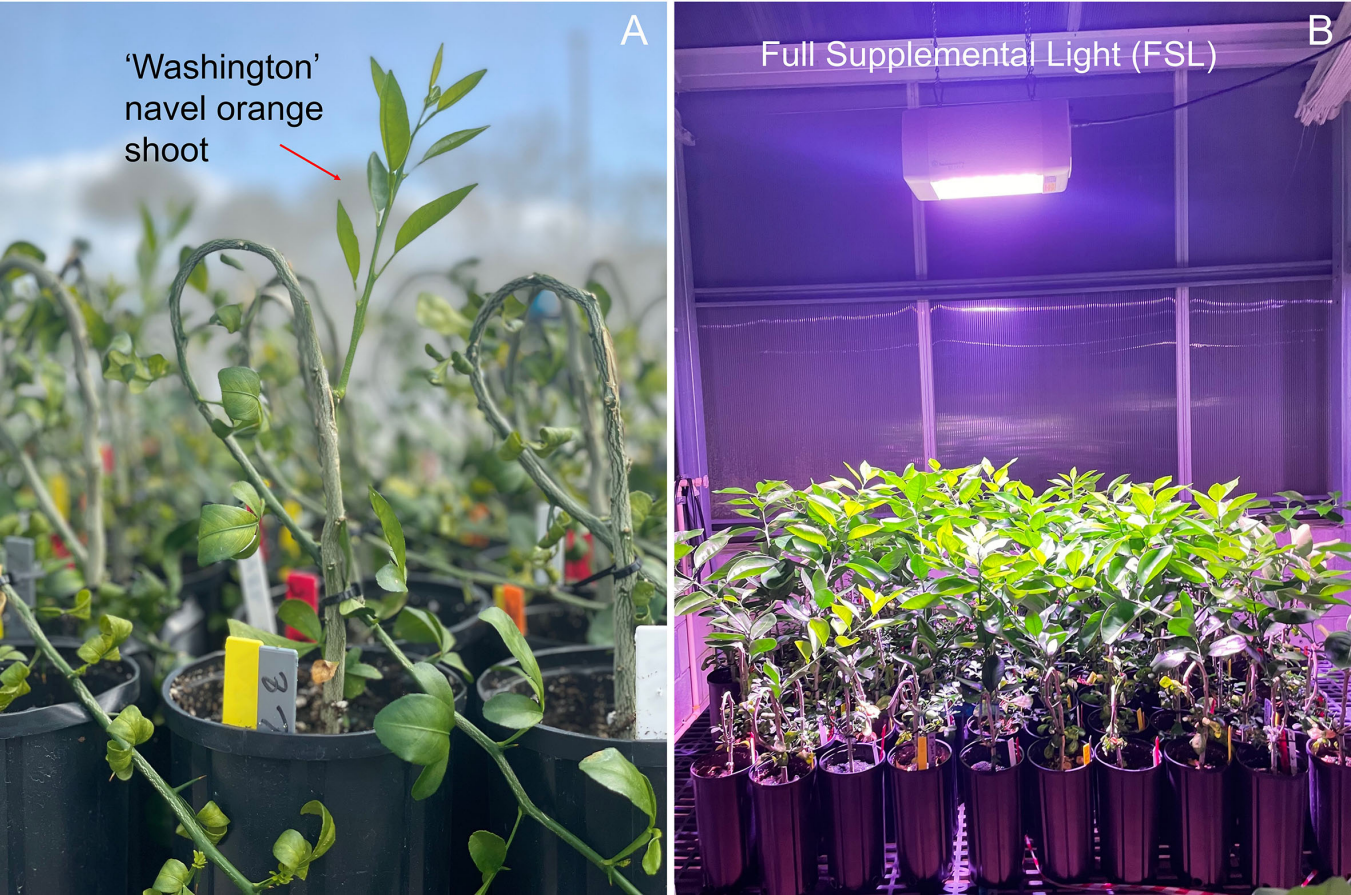
Shoot growth of grafted ‘Washington’ navel orange plants at 9 weeks after budding - wab **(A)** and at 12 wab under full supplemental light (FSL) **(B)** in a temperature-controlled greenhouse.

After budbreak, the length of shoots originating from ‘Washington’ navel buds was monitored every three weeks from 6 to 12 wab (**Table 2**). By 6 wab, significant interactions were found between light × PGR (p ≤ 0.001) and light × rootstock × PGR (p ≤ 0.05). The combination FSL × BA induced the most shoot elongation (7.6 cm) at 6 wab, while NoSL × NoBA promoted it the least (2.4 cm). FSL × NoBA improved shoot elongation more (5.8 cm) than BWSL × NoBA and BWSL-FSL × NoBA (4.1 and 3.5 cm, respectively). Considering all main factors, the combination BWSL-FSL × Carrizo × BA resulted in the longest shoot length among all other interactions at 6 wab (9.0 cm). This was notably longer than shoots resulting from the interaction of NoSL × Rubidoux × NoBA, which averaged 1.9 cm. By 9 wab (**Figure 3A**), a significant interaction was observed only between rootstock and PGR (p ≤ 0.001). In that case, Carrizo × BA showed the highest average shoot length (16.6 cm), differing from all other combinations, particularly those with Rubidoux (7.4–8.2 cm).

**Table 2:** Shoot length and shoot growth rate of ‘Washington’ navel orange nursery plants grafted on Carrizo citrange and Rubidoux trifoliate orange rootstocks grown under different supplemental LED lights and 6-benzylaminopurine (BA) at 6, 9, and 12 weeks after budding (wab) during the winter months.

Factor	Shoot length (cm)			Shoot growth
	6 wab	9 wab	12 wab	rate (cm day^-1^)
Light				
NoSL	4.5 c	10.9 b	22.4 c	0.43 c
FSL	6.7 a	12.6 a	32.7 a	0.62 a
BWSL	5.5 b	11.4 ab	27.7 b	0.53 b
BWSL-FSL	5.4 b	10.2 b	29.4 b	0.57 ab
Rootstock				
Carrizo	6.3 a	14.8 a	36.0 a	0.71 a
Rubidoux	4.8 b	7.8 b	20.1 b	0.36 b
PGR				
NoBA	4.0 b	10.2 b	24.1 b	0.48 b
BA	7.1 a	12.4 a	32.0 a	0.59 a
Light × Rootstock				
NoSL × Carrizo	5.0	14.6	29.0 c	0.57 c
NoSL × Rubidoux	4.1	7.2	15.8 e	0.28 e
FSL × Carrizo	7.8	16.5	42.2 a	0.82 a
FSL × Rubidoux	5.7	8.7	23.1 d	0.41 d
BWSL × Carrizo	6.4	14.7	34.9 b	0.68 b
BWSL × Rubidoux	4.7	8.1	20.5 d	0.38 d
BWSL-FSL × Carrizo	6.2	13.3	37.8 ab	0.75 ab
BWSL-FSL × Rubidoux	4.7	7.1	21.0 d	0.39 d
Light × PGR				
NoSL × NoBA	2.4 d	9.7	18.0	0.37
NoSL × BA	6.7 ab	12.1	26.8	0.48
FSL × NoBA	5.8 b	11.6	30.0	0.58
FSL × BA	7.6 a	13.5	35.3	0.66
BWSL × NoBA	4.1 c	10.4	23.9	0.59
BWSL × BA	6.9 ab	12.3	31.5	0.47
BWSL-FSL × NoBA	3.5 c	8.9	24.5	0.50
BWSL-FSL × BA	7.4 ab	11.4	34.3	0.64
Rootstock × PGR				
Carrizo × NoBA	4.5	13.0 b	31.8	0.65
Carrizo × BA	8.1	16.6 a	40.1	0.76
Rubidoux × NoBA	3.4	7.4 c	16.4	0.31
Rubidoux × BA	6.1	8.2 c	23.8	0.42
Light × Rootstock × PGR				
NoSL × Carrizo × NoBA	2.9 gh	13.0	24.5 e˗h	0.51
NoSL × Rubidoux × NoBA	1.9 h	6.4	11.6 j	0.63
NoSL × Carrizo × BA	7.0 a˗d	16.3	33.6 b˗d	0.23
NoSL × Rubidoux × BA	6.3 a˗d	8.0	20.1 g˗i	0.33
FSL × Carrizo × NoBA	6.8 a˗d	15.1	40.5 ab	0.80
FSL × Rubidoux × NoBA	4.8 d˗g	8.2	19.5 g˗i	0.35
FSL × Carrizo × BA	8.7 ab	17.9	43.9 a	0.84
FSL × Rubidoux × BA	6.6 a˗d	9.3	26.7 d˗g	0.48
BWSL × Carrizo × NoBA	4.9 d˗f	12.3	31.5 c˗e	0.63
BWSL × Rubidoux × NoBA	3.3 f˗h	8.0	16.3 ij	0.31
BWSL × Carrizo × BA	7.8 a˗c	16.5	38.4 a˗c	0.73
BWSL × Rubidoux × BA	6.0 b˗d	8.2	24.6 e˗h	0.44
BWSL-FSL × Carrizo × NoBA	3.4 f˗h	10.9	30.9 d˗e	0.65
BWSL-FSL × Rubidoux × NoBA	3.6 e˗g	7.0	18.0 h˗j	0.34
BWSL-FSL × Carrizo × BA	9.0 a	15.6	44.6 a	0.85
BWSL-FSL × Rubidoux × BA	5.7 c˗e	7.3	23.9 f˗h	0.43
p-value				
Light	***	***	***	***
Rootstock	***	***	***	***
PGR	***	***	***	***
Light × Rootstock	0.632	0.309	*	*
Light × PGR	***	0.912	0.169	0.570
Rootstock × PGR	0.320	***	0.582	0.954
Light × Rootstock × PGR	*	0.389	*	0.122

NoSL, no supplemental light from 0 to 12 wab; FSL, full supplemental light from 0 to 12 wab; BWSL, blue and white supplemental light from 0 to 12 wab; BWSL-FSL, blue and white supplemental light from 0 to 6 wab changing to full supplemental light from 6 to 12 wab. NoBA, no 6-benzylaminopurine; BA, 6-benzylaminopurine. Different letters within columns indicate significant differences between light treatments, rootstock cultivars, and plant growth regulators at each assessment according to Tukey’s honestly significant difference (HSD) test (α = 0.05). Level of significance: *, p ≤ 0.05; ***, p ≤ 0.001.

Similarly, significant interactions were noticed at the end of the experiment (12 wab), including light × rootstock (p ≤ 0.05) and light × rootstock × PGR (p ≤ 0.05). At that time (**Figure 3B**), the combination FSL × Carrizo promoted the longest shoot length (42.2 cm), while NoSL × Rubidoux resulted in the shortest (15.8 cm). Among the three-way interactions, FSL × Carrizo × BA and BWSL-FSL × Carrizo × BA resulted in the longest shoots (43.9–44.6 cm), while NoSL × Rubidoux × NoBA had the shortest (11.6 cm). A significant (p ≤ 0.05) interaction between light and rootstock was found for the shoot growth rate. The combination FSL × Carrizo had the highest growth rate (0.82 cm day^-1^), higher than most other combinations, except BWSL-FSL × Carrizo (0.75 cm day^-1^). The lowest growth rate was recorded for NoSL × Rubidoux (0.28 cm day^-1^).

### 3.3 Other vegetative growth and physiological traits

At the end of the experiment, additional plant responses were measured, including rootstock and scion stem diameters, total scion leaf area, scion dry biomass, number of nodes, internode length, chlorophyll index, and leaf surface temperature (**Table 3**). Significant interactions were observed between light × rootstock (p ≤ 0.001) and light × PGR (p ≤ 0.05) for rootstock stem diameter. The FSL × Carrizo combination exhibited the largest rootstock diameter (7.80 mm), contrasting with NoSL × Rubidoux (5.27 mm). Other combinations resulted in intermediate rootstock diameters. FSL, independent of the PGR levels, increased the rootstock diameter (6.67–6.77 mm) compared to the other light treatments. For the scion diameter, FSL × Carrizo induced the largest values (4.37 mm), while NoSL × Rubidoux had the lowest (2.59 mm). Carrizo exhibited a significant positive response to BA supplementation, resulting in a 7% increase in scion stem diameter than NoBA. Compared with Rubidoux, Carrizo had a remarkable 57% increase for this variable.

**Table 3:** Horticultural assessments of ‘Washington’ navel orange nursery plants grafted on Carrizo citrange and Rubidoux trifoliate orange rootstocks grown under different supplemental LED lights and 6-benzylaminopurine (BA) 12 weeks after budding (wab) in a greenhouse during the winter months.

Factor	Rootstock stem diameter (mm)	Scion stem diameter (mm)	Total scion leaf area (cm^2^)	Scion dry biomass (g)	Number of nodes	Internode length (mm)	Chlorophyll index	Leaf surface temperature (°C)
Light								
NoSL	6.13 c	3.23 c	244.5 c	2.47 d	8.0 c	13.2 c	78.5 a	21.7 c
FSL	6.72 a	3.60 a	455.6 a	5.17 a	12.4 a	24.1 a	70.9 b	22.9 a
BWSL	6.49 b	3.38 b	333.7 b	3.45 c	10.2 b	18.5 b	79.3 a	22.5 b
BWSL-FSL	6.51 b	3.36 b	370.7 b	3.98 b	11.6 ab	21.8 a	77.5 a	23.0 a
Rootstock								
Carrizo	7.44 a	4.07 a	506.9 a	5.57 a	13.3 a	26.9 a	79.5 a	22.7 a
Rubidoux	5.48 b	2.71 b	195.3 b	1.97 b	7.7 b	11.9 b	73.6 b	22.4 b
PGR								
NoBA	6.42 b	3.31 b	327.2 b	3.47 b	9.4 b	16.9 b	75.3 b	22.5
BA	6.62 a	3.48 a	375.0 a	4.06 a	11.7 a	21.9 a	77.7 a	22.5
Light × Rootstock								
NoSL × Carrizo	6.99 c	3.87 b	333.7 c	3.56 c	9.7 c	17.5 c	80.8	21.8
NoSL × Rubidoux	5.27 e	2.59 d	155.3 e	1.38 f	6.2 e	8.9 e	76.1	21.6
FSL × Carrizo	7.80 a	4.37 a	677.8 a	7.87 a	16.3 a	34.5 a	73.7	23.2
FSL × Rubidoux	5.63 d	2.82 c	233.4 d	2.47 d	8.6 cd	13.9 cd	68.1	22.7
BWSL × Carrizo	7.42 b	3.98 b	477.0 b	4.99 b	12.8 b	25.4 b	83.0	22.6
BWSL × Rubidoux	5.55 d	2.78 c	190.4 de	1.91 e	7.6 de	11.6 de	75.6	22.4
BWSL-FSL × Carrizo	7.53 b	4.06 b	539.1 b	5.85 b	14.5 ab	30.1 ab	80.5	23.1
BWSL-FSL × Rubidoux	5.48 de	2.66 cd	202.3 de	2.11 de	8.6 cd	13.5 cd	74.4	22.9
Light × PGR								
NoSL × NoBA	6.08 d	3.15	229.6 e	2.27	7.2 e	11.5 d	78.1 ab	21.7
NoSL × BA	6.19 cd	3.30	259.4 de	2.67	8.7 de	14.8 c	78.9 ab	21.7
FSL × NoBA	6.67 a	3.54	455.8 a	5.02	11.9 ab	23.4 ab	69.2 c	22.9
FSL × BA	6.77 a	3.65	455.4 a	5.32	13 ab	24.9 ab	72.7 c	23.0
BWSL × NoBA	6.40 bc	3.28	316.2 cd	3.15	9.1 de	16.2 c	76.8 b	22.5
BWSL × BA	6.57 ab	3.49	351.2 bc	3.75	11.3 bc	20.8 b	81.8 a	22.5
BWSL-FSL × NoBA	6.30 cd	3.25	307.3 cd	3.45	9.3 cd	16.6 c	77.2 b	23.2
BWSL-FSL × BA	6.72 a	3.47	434.1 ab	4.51	13.8 ab	26.9 a	77.7 b	22.9
Rootstock × PGR								
Carrizo × NoBA	7.31	3.93 b	469.1	5.12	11.8 b	23.6	78.8	22.7
Carrizo × BA	7.57	4.21 a	544.7	6.02	14.9 a	30.1	80.2	22.6
Rubidoux × NoBA	5.41	2.69 c	185.3	1.83	6.9 d	10.3	71.8	22.4
Rubidoux × BA	5.56	2.74 c	205.4	2.11	8.5 c	13.6	75.3	22.4
p-value								
Light	***	***	***	***	***	***	***	***
Rootstock	***	***	***	***	***	***	***	***
PGR	***	***	***	***	***	***	***	0.523
Light × Rootstock	***	*	*	***	***	***	0.462	0.077
Light × PGR	*	0.409	*	0.520	***	**	*	0.127
Rootstock × PGR	0.120	**	0.421	0.624	*	0.535	0.101	0.717
Light × Rootstock × PGR	0.247	0.567	0.310	0.203	0.104	0.653	0.171	0.931

NoSL, no supplemental light from 0 to 12 wab; FSL, full supplemental light from 0 to 12 wab; BWSL, blue and white supplemental light from 0 to 12 wab; BWSL-FSL, blue and white supplemental light from 0 to 6 wab changing to full supplemental light from 6 to 12 wab. NoBA, no 6-benzylaminopurine; BA, 6-benzylaminopurine. Different letters within columns indicate significant differences between light treatments, rootstock cultivars, and plant growth regulators at each assessment according to Tukey’s honestly significant difference (HSD) test (α = 0.05). Level of significance: *, p ≤ 0.05; **, p ≤ 0.01; ***, p ≤ 0.001.

The total leaf area also varied among the evaluated factors, with significant interactions for light × rootstock (p ≤ 0.05) and light × PGR (p ≤ 0.05). The FSL × Carrizo combination exhibited the largest leaf area (678 cm^2^), followed by BWSL × Carrizo and BWSL-FSL × Carrizo (477–539 cm^2^). The smallest leaf areas were observed for NoSL × Rubidoux (155 cm^2^). Among the light × PGR interactions, regardless of the PGR level, FSL induced the largest leaf area (455–456 cm²), while NoSL resulted in the smallest (230–259 cm²).

The light × rootstock interaction had a high effect on scion dry biomass (p ≤ 0.001). Plants grafted on Carrizo grown under FSL produced the greatest biomass (7.87 g), which was higher than that of Rubidoux grown without supplemental light (1.38 g), a 5.7-fold increase. Variations in the number of nodes and internode length were also detected across the factor levels and their interactions. Light interacted significantly with rootstock and PGR levels (p ≤ 0.01) for both the number of nodes and internode length. FSL was particularly effective in promoting nodes in the grafted shoots, notably in Carrizo (16 nodes, 68% more than NoSL) and with BA application (13 nodes, 81% more than NoSL). Rubidoux rootstock consistently showed fewer nodes, averaging 9 nodes after FSL supplementation. Significant rootstock × PGR interactions were observed, with Carrizo × BA resulting in 15 nodes, compared to a range of 7–12 nodes in other combinations. A similar trend was observed for internode length, where FSL was most effective in elongating shoots in both Carrizo (34.5 mm, 97% increase compared to NoSL) and Rubidoux (13.9 mm, 57% increase compared to NoSL), regardless of PGR levels (24.2 mm on average).

The study also identified a significant interaction between light and PGR (p ≤ 0.05) for the chlorophyll index, indicated by SPAD values, which serve as a relative indicator of chlorophyll content. Plants grown under BWSL × BA showed the highest index (81.8), similar to those without light supplementation (78.1–78.9) but differing from all other combinations (69.2–77.7). Although no significant interactions were found between the factors tested for the leaf surface temperature, differences were noted among the light and rootstock main factors. FSL increased leaf temperature by 5.7% compared to NoSL, and plants grafted on Carrizo showed an average increase of 0.3 °C in leaf temperature compared to Rubidoux.

**Figure 4:**
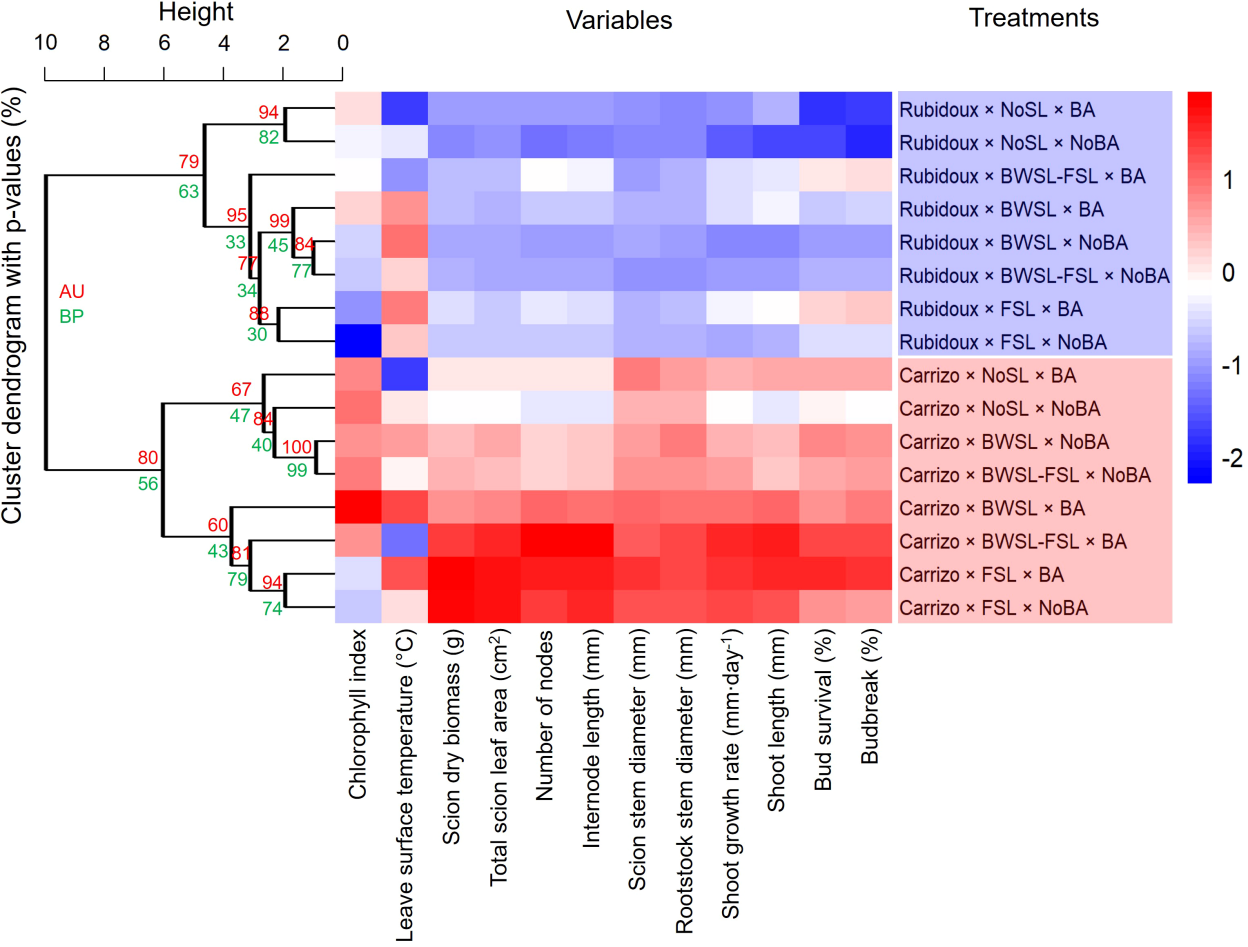
Hierarchical clustering and heatmap of horticultural variables for ‘Washington’ navel orange plants grafted onto Carrizo citrange and Rubidoux trifoliate orange rootstocks, grown under varied supplemental light (NoSL, no supplemental light from 0 to 12 weeks after budding (wab); FSL, full supplemental light from 0 to 12 wab; BWSL, blue and white supplemental light from 0 to 12 wab; BWSL-FSL, BWSL from 0 to 6 wab changing to FSL from 6 to 12 wab). Plant growth regulator treatments included NoBA (control) and BA (6-benzylaminopurine) brushed on buds 4 wab. All variables were assessed at 12 wab in a temperature-controlled greenhouse during the winter months. Heatmap colors indicate standardized values, with dark red presenting the highest values and dark blue the lowest. Clusters in the dendrogram were evaluated using Approximately Unbiased (AU) and Bootstrap Probability (BP) p-values, with AU values closer to 100% considered more robust.

The hierarchical clustering and heatmap analysis revealed clear trends in the effect of light, rootstock, and PGR on budbreak and shoot growth responses (**Figure 4**). Two major clusters were identified, separating Carrizo and Rubidoux rootstocks. Carrizo combinations consistently exhibited higher values across most growth response variables, including budbreak, bud survival, shoot length, shoot growth rate, and total leaf area, compared to Rubidoux combinations. Within each rootstock cluster, treatments with BA generally grouped together, indicating a consistent positive effect of the PGR on bud development and growth. For Carrizo, the combinations of BWSL-FSL × BA and FSL × BA showed the highest standardized scores across multiple responses, reflecting the strongest growth performance. In contrast, combinations without BA or under NoSL tended to cluster with lower growth performance, particularly in Rubidoux, which exhibited lower chlorophyll index, leaf area, and budbreak rates. The dendrogram with statistical support (AU p-values) confirmed that clusters of combinations with similar growth responses were robust, reinforcing that both light spectrum and BA application contributed to the observed differences.

### 3.4 Temperature and daily light integral

Daily air temperatures inside the greenhouse were monitored throughout the experiment (**Table 4**; **Supplementary Figure 1**). Analysis of maximum, minimum, and mean temperatures across this period revealed no variations between the tested LED spectra or interactions between light and time (weeks after budding, wab). However, differences were observed when examining the effects of wab levels. During the first six weeks of the experiment (0–6 wab), greenhouse average temperatures peaked at a maximum of 29.2 °C, with a minimum of 22.5 °C and a mean of 25.1 °C. Subsequently, temperatures decreased by approximately 1.1 °C as winter approached (6–9 wab). In the final period (9–12 wab), temperatures slightly increased compared to the previous period by 0.2–0.5 °C. Calculation of the daily light integral showed a significant interaction between light and wab (p ≤ 0.001). Notably, treatments receiving FSL and BWSL-FSL exhibited the highest daily light integrals during the last two periods, averaging over 16 mol m^-2^day^-1^ (**Table 4**; **Supplementary Figure 1**). In contrast, treatments without supplemental light (NoSL) had the lowest averages, ranging from 7.0 to 8.2 mol m^-2^day^-1^.

**Table 4:** Average air temperature (maximum - Max., minimum - Min., and mean) and daily light integral inside the greenhouse used for growing ‘Washington’ navel orange nurseries grafted onto Carrizo citrange and Rubidoux trifoliate orange rootstocks, subjected to different supplemental LED lights and 6-benzylaminopurine (BA) 12 weeks after budding (wab) during the winter months.

Factor	Air temperature (°C)			Daily light integral (mol m^2^ day)
	Max.	Min.	Mean	
Light				
NoSL	28.8	21.8	24.4	7.5 c
FSL	28.8	21.7	24.5	16.3 a
BWSL	28.6	21.8	24.4	11.5 b
BWSL-FSL	28.9	21.7	24.5	15.3 a
Time after budding				
0–6 wab	29.2 a	22.5 a	25.1 a	10.3 c
6–9 wab	28.3 c	21.3 c	23.9 c	13.1 b
9–12 wab	28.8 b	21.5 b	24.3 b	14.5 a
Light × Time after budding				
NoSL × 0–6 wab	30.0	22.6	25.1	7.0 g
NoSL × 6–9 wab	28.8	21.3	23.8	7.4 g
NoSL × 9–12 wab	29.6	21.5	24.3	8.2 fg
FSL × 0–6 wab	30.2	22.4	25.2	13.9 bc
FSL × 6–9 wab	28.7	21.3	23.9	16.8 a
FSL × 9–12 wab	29.5	21.5	24.3	18.0 a
BWSL × 0–6 wab	29.7	22.5	25.1	9.7 e-g
BWSL × 6–9 wab	29.2	21.4	24.0	11.7 c-e
BWSL × 9–12 wab	29.1	21.6	24.3	13.0 cd
BWSL-FSL × 0–6 wab	29.7	22.4	25.0	10.7 d-f
BWSL-FSL × 6–9 wab	29.3	21.3	24.0	16.6 ab
BWSL-FSL × 9–12 wab	29.7	21.5	24.4	18.7 a
p-value				
Light	0.581	0.455	0.526	***
wab	***	***	***	***
Light × wab	0.200	0.963	0.388	***

NoSL, no supplemental light from 0 to 12 wab; FSL, full supplemental light from 0 to 12 wab; BWSL, blue and white supplemental light from 0 to 12 wab; BWSL-FSL, blue and white supplemental light from 0 to 6 wab changing to full supplemental light from 6 to 12 wab. wab, weeks after budding. Different letters within columns indicate significant differences between light treatments, rootstock cultivars, and plant growth regulators at each assessment according to Tukey’s honestly significant difference (HSD) test (α = 0.05). Level of significance: ***, p ≤ 0.001.

## 4 Discussion

This study revealed that supplemental light (LED), rootstock genotype, and the application of synthetic cytokinin (BA) each influenced the vegetative growth of ‘Washington’ navel orange nursery plants during the short days of winter, with responses varying according to the specific factor and horticultural response variable assessed. Our results demonstrate that broad-spectrum light, corresponding to the FSL treatment (comprising blue at 450 nm, red at 660 nm, far-red at 735 nm, and white light at 446–625 nm), substantially improved budbreak and bud survival rates by 26% and 25%, respectively, compared to control plants (NoSL). These increases are critical for grafting success, as they promote vigorous growth and greater uniformity among nursery plants, reducing plant culling and improving overall production efficiency. In addition, the combination of blue light at 450 nm and white light (BWSL) at 446–625 nm, either alone or combined with FSL (BWSL-FSL), also enhanced budbreak, albeit to a lesser extent (17% and 24%, respectively). This finding reinforces the essential role of broad and blue/white light spectra in mimicking natural light conditions and stimulating bud development, consistent with the outcomes of previous studies, which showed that broad-spectrum light significantly increased vegetative budbreak in grafted citrus during winter (Bowman and Albrecht, 2021; Bisi et al., 2024), while blue and white light have been shown to stimulate shoot induction in *in vitro*–cultured apical buds of *Ficus carica* L. var. Black Jack (Parab et al., 2021), supporting the idea that spectral composition can modulate bud growth. Taken together, these results suggest that the integration of blue and white wavelengths, whether as part of a full spectrum or targeted combinations, represents a strategy for maximizing budbreak and early growth performance in citrus nurseries under low-light seasonal conditions.

Previous studies have shown that specific light spectra, particularly those produced by LEDs, can efficiently promote bud outgrowth by influencing hormonal pathways involved in plant growth (Lau and Deng, 2010; Schneider et al., 2019). These studies indicate that optimally adjusted light intensity has been associated with cytokinin production, which is essential for promoting bud development. In contrast, a low red-to-far-red light ratio triggers the production of abscisic acid, which inhibits bud outgrowth (Schneider et al., 2019). This illustrates the complex regulatory mechanisms that control bud development, emphasizing the importance of light quality on plant growth responses. For instance, extended daylength using LEDs as a light source increased the percentage of budbreak of ‘Valencia’ sweet orange grafted onto different rootstocks by 13% compared to natural light alone (Bowman and Albrecht, 2021). Red LED increased bud outgrowth in pot and garden chrysanthemum (*Chrysanthemum morifolium* Ramat.), while blue, combined with far-red light, resulted in a reduced budbreak rate (Dierck et al., 2017). These findings are consistent with ours, where FSL using red light at 660 nm, blue at 450 nm, far-red at 735 nm, and white light at 446–625 nm led to the highest percentage of bud outgrowth. Therefore, implementing supplemental light in citrus nurseries can lead to a more consistent and earlier onset of budbreak, ultimately enhancing production efficiency and profitability during winter.

Plants exposed to supplemental light, particularly FSL, showed increases in shoot length (1.46-fold) and internode length (1.83-fold) compared to control plants. Daylength extension using full-spectrum LED also positively influenced shoot elongation in ‘Washington’ navel plants in a previous study (Bisi et al., 2024), although it did not have a significant effect on internode length. Blue light also increased shoot length by 1.23-fold and internode length by 1.40-fold in our study. These findings are consistent with other studies on ornamental plants, where extending the photoperiod with blue light promoted stem and internode elongation in chrysanthemum (Jeong et al., 2014) and petunia (*Petunia × hybrida* Vilm.) plants (Fukuda et al., 2016).

Similar to supplemental light, the application of the synthetic plant growth regulator BA improved the budbreak of the grafted buds, in line with previous reports identifying this chemical as a positive regulator of axillary bud outgrowth (Ni et al., 2017). BA application resulted in an average budbreak of 77%, a 9% improvement compared to the untreated control (NoBA). This cytokinin was also an effective agent for stimulating lateral branch development in apple (*Malus × domestica* Borkh.) trees (Wertheim and Estabrooks, 1994; McArtney and Obermiller, 2015) and other species, such as tea (*Terminalia bellerica* Roxb.) plants (Mandal, 2021).

FSL, regardless of PGR levels, consistently produced larger stem diameters in both rootstocks, with the effect being most pronounced with Carrizo. This aligns with previous research showing that supplemental artificial light stimulates cell division and expansion across various plant organs (Okello et al., 2016), resulting in thicker stems and enhanced growth (Riikonen et al., 2016). The weaker response of Rubidoux to light supplementation suggests that it was more dormant at the time of experimentation than Carrizo. This is likely associated with the deciduous nature of Rubidoux (Yelenosky, 1985; Primo-Millo and Agustí, 2020), which is a pure trifoliate orange (*P. trifoliata*), in contrast to Carrizo, which is a hybrid of sweet orange (*C.* × *sinensis*) with trifoliate orange. Hierarchical clustering analysis further emphasized this trend, clearly separating Carrizo and Rubidoux and revealing consistent performance differences across all measured growth traits. These findings demonstrate that Carrizo performance is both consistent and robust across a wide range of growth indicators. The smallest stem diameters were recorded for Rubidoux under NoSL and BWSL-FSL, supporting the idea that certain rootstocks may have a reduced capacity to benefit from supplemental light (Martínez-Cuenca et al., 2016). It suggests that rootstock genotype is a critical determinant of the plant’s ability to utilize supplemental light under winter dormancy, a concept supported by studies on rootstock effects in citrus (Brar and Spann, 2014; Bowman and Albrecht, 2021; Bisi et al., 2024).

BA application further enhanced stem growth, particularly in Carrizo, which responded with a 7% increase in scion diameter compared to the control. This corroborates previous studies that demonstrated the positive effects of cytokinin in promoting vegetative growth. For instance, BA enhanced budbreak and shoot elongation in paradormant apple buds, reinforcing its potential to improve growth and yield under challenging conditions (McArtney and Obermiller, 2015). Within each rootstock in the hierarchical cluster analysis, treatments including BA application were consistently grouped together, indicating the cytokinin’s broad stimulatory effect on bud outgrowth and vegetative growth.

FSL had a pronounced effect on scion leaf area and dry biomass, with Carrizo exhibiting the highest values, suggesting that this light spectrum has significant potential to enhance plant radiation use efficiency in citrus nurseries. This aligns with previous studies by Legendre and Van Iersel (2021) and Park and Runkle (2017), who reported that supplemental light, particularly with red and far-red spectra, can stimulate leaf expansion and increase total leaf area, thereby improving photosynthetic capacity and growth. However, while FSL promoted leaf size, it also resulted in the lowest chlorophyll index, particularly in buddings originating from Rubidoux. This finding suggests that the increase in leaf growth may not always be fully accompanied by a proportional increase in leaf greenness. Although our study did not measure chlorophyll synthesis directly, previous work has shown that certain light spectra, particularly red-enriched light, can be associated with reduced chlorophyll concentration despite the increased leaf area (Meng and Runkle, 2019), which may help explain the observed pattern in our study. This phenomenon is consistent with Zou et al. (2019), who demonstrated that while the addition of far-red light enhanced leaf area and improved light interception, it also caused a downregulation in chlorophyll content, resulting in reduced leaf absorbance and lower maximum leaf photosynthetic rate. This trade-off shows that the balance between light intensity and quality must be carefully managed to optimize both leaf growth and photosynthetic efficiency, particularly in environments where excessive light can lead to physiological stress.

In contrast to FSL, BWSL combinations did not reduce the leaf chlorophyll index in plants, despite increasing the leaf area, confirming the importance of blue and white light in chlorophyll synthesis. Blue light has been shown to increase leaf area and shoot elongation as well as chloroplast development in cucumber (*Cucumis sativus* L.) (Wang et al., 2015), as they stimulate photosynthetic processes and enhance efficiency. Gao et al. (2020) demonstrated that blue and white light improved the photosynthetic efficiency and growth of Welsh onions (*Allium fistulosum* L.). These findings suggest that both light quality (i.e., blue and white spectra) and intensity, in combination with BA, can synergistically influence chlorophyll biosynthesis and photosynthetic capacity in citrus plants.

FSL resulted in a moderate 5.7% increase in leaf surface temperature compared to the control. Although this difference is relatively small and may have limited physiological relevance, it indicates that LEDs, despite being more energy-efficient than traditional sources such as high-pressure sodium (HPS), can still contribute to slight leaf heating, particularly in controlled greenhouse environments where heat can accumulate (Nelson and Bugbee, 2015). A slight but significant increase (0.3 °C) in leaf temperature was observed in plants grafted on Carrizo compared to Rubidoux, likely because of the stronger growth response of Carrizo and therefore greater proximity of the leaves to the light source. While temperature dynamics were primarily driven by seasonal variations, the DLI was modulated by both light spectra and the progression of the experiment. Indeed, supplemental light (i.e., FSL and BWSL-FSL) significantly increased DLI compared to the NoSL control, particularly during the later growth stages (6–9 and 9–12 weeks after budding). These results demonstrate that supplemental lighting effectively offsets the reduction in natural light during winter, thereby sustaining photosynthetic activity and supporting plant growth (Tewolde et al., 2016). Additionally, the interaction between light and plant developmental stage confirms the importance of optimizing light management as plants transition through different growth phases. The higher DLIs observed in the later growth stages of the experiment were likely crucial in sustaining extended photosynthetic capacity and overall plant vigor during the critical phases of development.

The complex interactions between light quality, rootstock genotype, and PGR observed in this study show the potential to strategically combine these factors to optimize citrus nursery growth, particularly during the short, low-light days of winter. Our results demonstrate that both light spectrum and exogenous cytokinin application can significantly enhance key growth responses, but that the magnitude of these effects is strongly influenced by rootstock genotype. The heatmap analysis reinforced this conclusion, revealing that the combination of supplemental light, particularly broad-spectrum or blue + white combinations, and BA yielded the most pronounced improvements in Carrizo, while Rubidoux exhibited comparatively smaller gains. Importantly, these benefits are not independent of environmental conditions, as demonstrated in our previous work, in which low greenhouse temperatures can restrict the positive impacts of supplemental light, confirming the need to improve both temperature and light regimes to maximize growth responses during winter (Bisi et al., 2024). This reinforces the importance of tailoring light, temperature, and PGR management practices to the specific physiological responses of each rootstock, rather than applying uniform strategies across genotypes. By integrating targeted light regimes with BA applications under controlled temperature conditions, nurseries can improve grafting success, promote more uniform growth, and accelerate readiness for field establishment, ultimately increasing production efficiency and profitability under challenging seasonal conditions.

## 5 Conclusions

Full-spectrum supplemental light (FSL) to extend daylength during the winter proved highly effective in improving budbreak and bud survival in ‘Washington’ navel orange, resulting in vigorous shoot growth. The combination of blue and white light changing to FSL (BMSL-FSL) was also effective, though at a lesser magnitude. Additionally, 6-benzylaminopurine (BA) enhanced budbreak potential and shoot development, particularly when combined with appropriate supplemental LED light. Carrizo citrange outperformed Rubidoux trifoliate orange in promoting bud growth and shoot elongation, demonstrating the importance of matching supplemental light and cytokinin treatments with rootstock selection to optimize plant responses with economic benefit. These results demonstrate that the strategic use of FSL, BWSL, and BA, in combination with the right rootstock, may improve the consistency, vigor, and productivity of citrus nurseries. This approach has important implications for improving nursery operations, particularly during the winter months when natural light is scarce.

## Data Availability

The original contributions presented in the study are included in the article/supplementary material. Further inquiries can be directed to the corresponding authors.
