# Supplementary material for: LED lighting and exogenous cytokinin enhance budbreak and winter growth of ‘Washington’ navel orange in the nursery
Source: Front Plant Sci. 2025 Dec 16;16:1735154. doi: 10.3389/fpls.2025.1735154 (PMC12748169; doi:10.3389/fpls.2025.1735154)
Supplement: Supplementary file 1 [file Table1.docx]

Supplementary Material


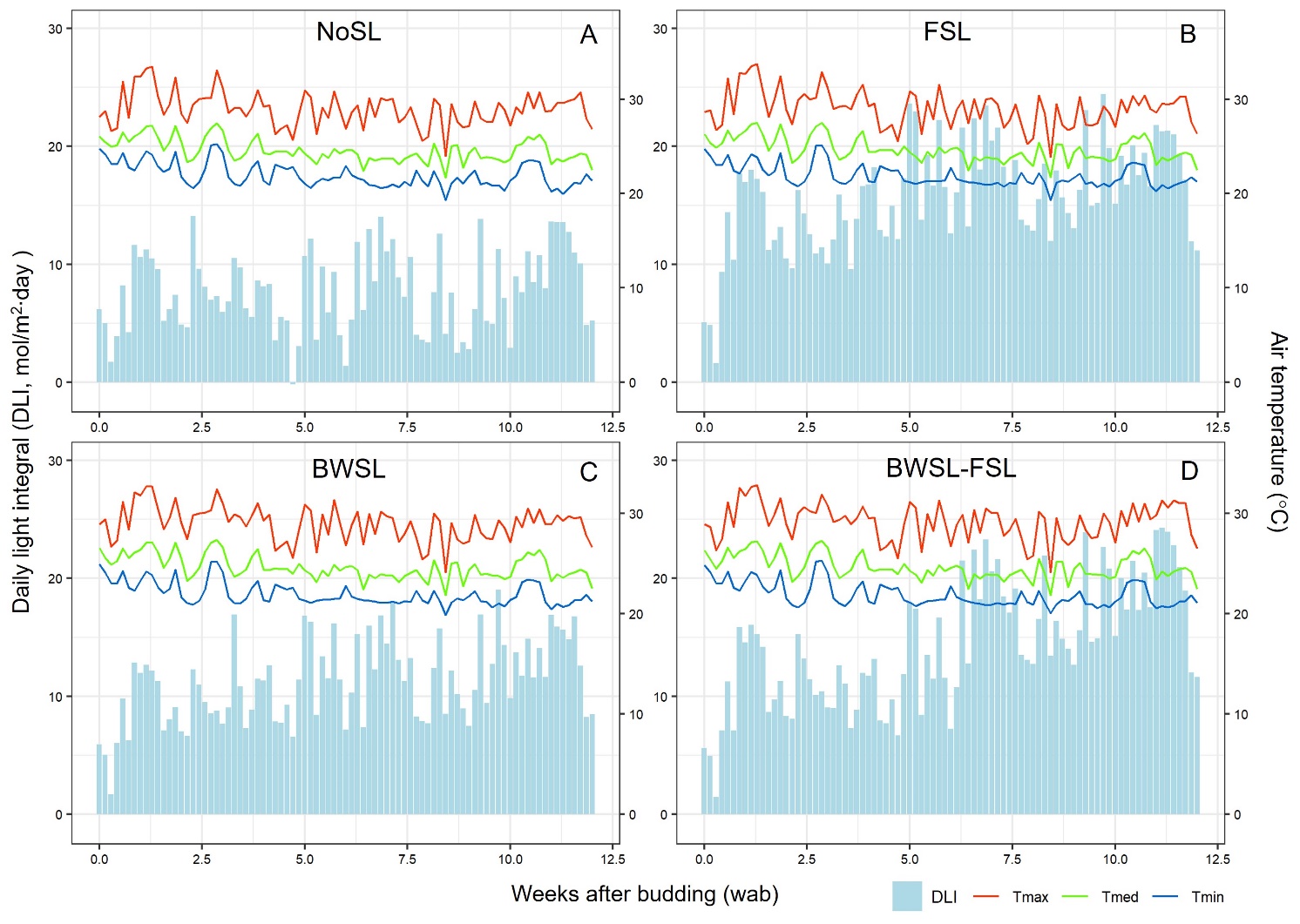


**Supplementary Figure 1.** Average maximum (Tmax), mean (Tmed), and minimum (Tmin) temperatures (°C) and daily light integrals (DLIs; mol·m⁻²·day⁻¹) recorded under each supplemental LED lighting treatment during winter in a temperature-controlled greenhouse. Treatments included: NoSL (no supplemental light from budding (0) to 12 weeks after budding, wab; A), FSL (full-spectrum supplemental light from 0 to 12 wab; B), BWSL (blue and white supplemental light from 0 to 12 wab; C), and BWSL-FSL (BWSL from 0 to 6 wab changing to FSL from 6 to 12 wab; D).
